# Diversity of *Colletotrichum* Species Causing Anthracnose in Chayote in Brazil, with a Description of Two New Species in the *C. magnum* Complex

**DOI:** 10.3390/jof10120847

**Published:** 2024-12-09

**Authors:** Willie Anderson dos Santos Vieira, Christiane Almeida da Costa, Josiene Silva Veloso, Waléria Guerreiro Lima, Kamila Câmara Correia, Sami Jorge Michereff, Danilo Batista Pinho, Marcos Paz Saraiva Câmara, Ailton Reis

**Affiliations:** 1 Departamento de Fitopatologia, Universidade de Brasília, Brasília 70910-900, Brazil; andersonvieira12@gmail.com (W.A.d.S.V.); danilopinho@unb.br (D.B.P.); 2 Departamento de Agronomia, Universidade Federal Rural de Pernambuco, Recife 52171-900, Brazil; eng.christiane.a.costa@gmail.com (C.A.d.C.); marcos.camara@ufrpe.br (M.P.S.C.); 3 AFYA, Faculdade de Ciências Médicas, Jaboatao do Guarapes 54410-100, Brazil; waleria.lima@afya.com.br; 4 Centro de Ciências Agrárias e da Biodiversidade, Universidade Federal do Cariri, Crato 63130-025, Brazil; kamila.correia@ufca.edu.br (K.C.C.); sami.michereff@ufca.edu.br (S.J.M.); 5 Laboratório de Fitopatologia, Embrapa Hortaliças, C. Postal 218, Brasilia 70250-970, Brazil

**Keywords:** *Sicyos edulis*, multilocus phylogeny, morphology, pathogenicity

## Abstract

Anthracnose caused by *Colletotrichum* species is the most important disease of chayote (*Sicyos edulis*) in Brazil. The etiology of chayote anthracnose has been assigned to the species *C. orbiculare*, an important plant pathogenic fungus also reported as the causal agent of anthracnose in other cucurbits worldwide. However, there is no recent survey of the *Colletotrichum* species causing anthracnose in chayote in Brazil. In this study, *Colletotrichum* isolates associated with anthracnose on the fruit and leaves of chayote, from various producing regions in Brazil, were collected and identified. Haplotype analysis based on sequences of the β-tubulin genomic region (*TUB2*) of 44 *Colletotrichum* isolates was carried out as a first measure of genetic diversity. A subset of 22 isolates were sequenced using the partial sequences of actin (*ACT*), glyceraldehyde-3-phosphate dehydrogenase (*GAPDH*), and the rDNA ITS (ITS) region. Maximum likelihood analysis was performed using the concatenated sequences. The multilocus sequence analysis revealed four previously described species, *Colletotrichum chrysophilum*, *C. menezesiae*, *C. plurivorum*, and *C. karsti,* and two novel species, named *C. cucurbitacearum* and *C. sicyi*. All species were able to induce typical symptoms of anthracnose in chayote fruits but varied in their aggressiveness. The species *C. menezesiae* and *C. sicyi* were the most aggressive, while *C. plurivorum* was the least aggressive. The species *C. orbiculare* was not found to cause chayote anthracnose in Brazil.

## 1. Introduction

The chayote [*Sicyos edulis* (Jacq.) Swartz; syn. *Sechium edule* (Jacq.) Swartz] is an herbaceous vegetable belonging to the Cucurbitaceae family [1]. The crop is commercially produced in tropical and subtropical areas of Brazil, Costa Rica, Italy, Mexico, Puerto Rico, Australia, New Zealand, Indonesia, China, India, and some African countries [2,3]. In Brazil, it is among the 10 most consumed vegetables [4]. Some species from Cucurbitaceae family, such as chayote, are commonly produced in relatively small quantities for local consumption and therefore do not usually appear on more significant production statistics, although they are important items in the diet of many Brazilian people [5].

**Figure 1:**
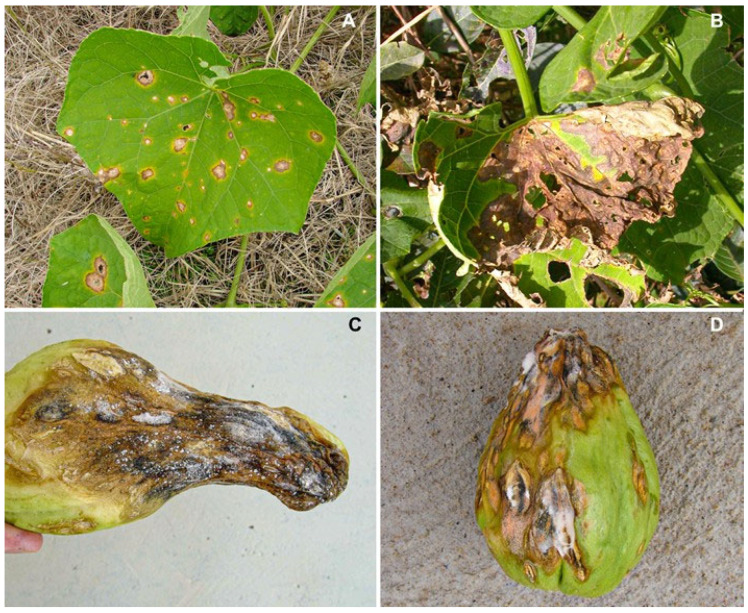
Anthracnose symptoms in chayote: circular to irregular lesions on leaves (**A**), foliar blight (**B**), fruit rotting (**C**), post-harvest lesions filled with fungus spore masses of pale-pink color (**D**).

Biotic diseases that cause severe yield loss can affect cucurbitaceous vegetables. Anthracnose, caused by species of the genus *Colletotrichum*, can be considered the most important fungal disease of chayote in Brazil. Chayote anthracnose occurs in all producing areas in Brazil, usually with high severity [1,6]. Anthracnose symptoms mainly occur on leaves and fruits in any stage of development. Foliar anthracnose begins as circular to irregular lesions (Figure 1A), which can coalesce and cause foliar blight (Figure 1B). On fruit and stems, lesions are elliptical, depressed and dark, turning a pale-pink color with the pathogen sporulation. Infections, when severe, cause falling leaves and fruit rotting (Figure 1C). The disease is also very common in post-harvest. Apparently, healthy fruit when harvested can develop anthracnose symptoms after a few days. In storage conditions, lesions may coalesce to form larger and depressed lesions, which in moist conditions are filled with fungus spore masses of pale-pink color (Figure 1D) [1,6,7].

The etiology of chayote anthracnose has been assigned to the species *C. orbiculare* (syn. *C. lagenarium*), which is an important pathogen also reported as the causal agent of anthracnose in other cucurbits such as cucumber (*Cucumis sativus* L.), melon (*Cucumis melo* L.), watermelon [*Citrullus lanatus* (Thunb.) Matsum. & Nakai], zucchini (*Cucurbita pepo* L.), squash (*Cucurbita maxima* Duchesne), and pumpkin (*Cucurbita moschata* Duchesne ex Poir). This species belongs to the *C. orbiculare* species complex and has been reported only in cucurbitaceous hosts, which may indicate a host specificity to this botanical family [8,9].

The genus *Colletotrichum* historically has been systematically confused and unstable. Using only morphological and phenotypic criteria has led researchers to errors in defining and identifying the species in this genus because of the small number of morphological features along with the high plasticity of these characteristics under different environmental conditions. Following the use of molecular data and standardized growth conditions, many *Colletotrichum* strains have been identified, epitypified, and grouped within an existing species complex [10,11,12,13,14,15,16].

The accurate identification and characterization of the diversity of *Colletotrichum* causing anthracnose in chayote is essential to understanding the epidemiology of the disease and to assist in the development of more efficient control strategies. However, there are no worldwide studies on the molecular characterization of *Colletotrichum* species causing chayote anthracnose using a wide collection of isolates representing different production regions. To date, two studies have been carried out in Brazil. Sussel 2005 [17] identified one isolate from chayote as *C. lagenarium* by using a species-specific primer pair from the ITS region. In a second study, only one isolate from chayote was identified as *C. brevisporum* by multilocus phylogeny of sequences of six genomic regions [18]. In addition, in a study that performed the epitypification of the *C. orbiculare* complex, no *Colletotrichum* isolate from chayote was characterized [8].

Therefore, the aim of this study was to identify the species of *Colletotrichum* causing chayote anthracnose in different production regions of Brazil based on multilocus phylogeny and to determine the pathogenicity and aggressiveness of representative isolates on chayote fruit.

## 2. Materials and Methods

### 2.1. Sampling and Fungal Isolation

Fruit and leaves of chayote showing anthracnose symptoms were collected in different producing regions of Brazil. This included samples from Pernambuco, Santa Catarina, Rio Grande do Sul, and Minas Gerais states and from the Distrito Federal. Tissue fragments of fruit and leaves (4–5 mm) with symptoms of anthracnose were surface disinfested in 70% ethanol for 30 s and 1% NaClO for 1 min and then rinsed in sterile distilled water for 30 s and dried on sterilized paper before culturing on potato dextrose agar (PDA) at 28 °C with a 12 h photoperiod provided by fluorescent light for 5 days. Subsequently, hyphal tips were transferred to new PDA plates. Some isolates were obtained directly from chayote fruit with sporulating lesions. Spore masses were picked off from the lesions with a sterilized metal handle and streaked on the surface of water agar. Single germinated spores were picked up with a sterilized needle and transferred to PDA plates after 2–3 days at 28 °C [19]. Forty-four isolates were obtained and morphologically identified as *Colletotrichum* spp. [20]. Pure cultures of the isolates were stored in sterilized water in microtubes. The isolates were deposited in the culture collection of the Universidade Federal Rural de Pernambuco, Coleção de Fungos Fitopatogênicos “Professora Maria Menezes” (CMM), Recife, Pernambuco, Brazil. The new species ex-type living cultures were also preserved in 15% glycerol and deposited in the culture collection of the Universidade Federal de Viçosa, Coleção Oswaldo Almeida Drummond (COAD), Viçosa, Minas Gerais, Brazil. Cultures in an inactive metabolic state were deposited at the Herbarium of the Universidade Federal de Viçosa (VIC).

### 2.2. Molecular Characterization

The isolates were cultured in Petri dishes of 90 mm diameter for 5 days at 28 °C with a photoperiod of 12 h. Aerial mycelium was scraped from the colony surface and genomic DNA was extracted by the protocol of Doyle and Doyle 1990 (San Diego, CA, USA) [21]. DNA samples were visualized in 1% agarose gel to check their quality. The DNA concentration measurements were carried out on a NanoVue Plus Spectrophotometer (GE Healthcare, Chicago, IL, USA).

The β-tubulin (*TUB2*) partial sequence was amplified and sequenced to estimate the genetic diversity of all isolates. Haplotypes were identified using DnaSP 4.0 [22]. Sequences were compared with the NCBI database using the BLAST tool to determine the *Colletotrichum* species complex to which each isolate belonged. A subset of 22 isolates representing genetic diversity and collecting regions was selected for multilocus analysis (Supplementary Table S1). The glyceraldehyde-3-phosphate dehydrogenase (*GAPDH*), actin (*ACT*), and rDNA-ITS (ITS) partial regions were also sequenced and amplified (Table 1).

The PCR amplifications for all genomic regions were performed in a final volume of 50 μL, containing 1 µL of DNA, 28.2 µL of water, 2.5 µL of each primer, 5 µL of 10× PCR buffer, 4 µL of MgCl2, 0.3 µL of Platinum Taq DNA Polymerase High Fidelity (Invitrogen™, ThermoFisher Scientific, Waltham, MA, USA), 1.5 µL DMSO², and 5.0 µL dNTP (Invitrogen™).

PCR reactions were carried out in a thermal cycler (MultiGene™ OptiMax; Labnet International, Edison, NJ, USA). The cycling parameters for *GAPDH* and *ACT* consisted of a denaturing step at 94 °C for 2 min, followed by 35 cycles at 94 °C for 45 s, 60 °C for 45 s, 72 °C for 1 min, and a final cycle at 72 °C for 10 min. For *TUB2*, they consisted of a denaturing step at 94 °C for 5 min, followed by 34 cycles at 94 °C for 30 s, 52 °C for 30 s, 72 °C for 1 min, and a final cycle at 72 °C for 10 min. For the ITS region, they consisted of a 3 min denaturing step at 95 °C, followed by 34 cycles at 95 °C for 1 min, 50 °C for 1 min, 72 °C for 1 min e 30 s, and a final cycle at 72 °C for 10 min. PCR products were purified using the AxyPrep™PCR CleanupKit (Axygen/Corning, San Diego, CA, USA) following the manufacturer’s instructions. The DNA sequencing was carried out by Macrogen Inc. (Seoul, Republic of Korea).

### 2.3. Phylogenetic Reconstruction and Species Recognition

Sequences from previous studies were retrieved from GenBank (Supplementary Table S1) in MEGA 7 [28]. Individual multiple-sequence alignments were estimated with the online version of MAFFT 7 [29,30] using the G-INS-i iterative refinement method. Alignments were concatenated in Sequence Matrix v. 1.8 [31]. Individual and concatenated alignments were used to build multilocus phylogenetic trees.

Maximum likelihood (ML) analyses were performed using IQ-TREE v. 2.1.2 [32], keeping identical sequences in the alignment. Model parameters were estimated for each partition using ModelFinder [33,34], allowing each partition to have its evolution rate. The best ML tree was found after 1000 iterations with a perturbation strength of 0.2. Branch supports were estimated using the approximate likelihood ratio test with Shimodaira–Hasegawa interpretation (SH-aLRT) [35] with 1000 bootstrap samples. Clades were well supported when the SH-aLRT bootstrap support was ≥ 80%.

Species were recognized utilizing the Genealogical Concordance Phylogenetic Species Recognition (GCPSR) criteria, as described by Taylor et al. 2000 [36] and Dettman et al. 2003 [37].

### 2.4. Phenotypical Characterization

Phenotypical characterization was carried out for isolates of novel species. Five-millimeter-diameter plugs were taken from the expanding margin of 7-day-old colonies and transferred to the center of PDA Petri dishes. Each isolate was placed on three replicate plates and incubated at 25 °C under continuous fluorescent light. Four radial measurements were taken from the edge of the plug to the margin of the colony every 24 h, with the first measurement taken after 48 h of incubation. The distance between the center and the marks was used to calculate the mycelial growth rate (mm/day). Cultures were kept under the same incubation conditions and colony characters were recorded from 7-day-old cultures.

To enhance conidiation, the isolates were grown on oatmeal agar (20% strength). Fungal structures were mounted in 10% lactic acid for microscopic observation. Hyphal appressoria were observed using slide cultures [38]. Photomicrographs were made with a DS-L3 digital camera attached to a Nikon Eclipse Ni-U transmitted light microscope using differential interference contrast illumination. Microscopic image capture and measurement were carried out using NIS-Elements. At least 30 measurements per structure were taken and summary statistics were calculated using the software Statistix 9 (Analytical Software, Tallahassee, FL, USA).

### 2.5. Pathogenicity and Aggressiveness Testing in Fruits

Thirteen isolates were tested for pathogenicity and aggressiveness on healthy chayote fruit. Fruits were washed in running water and disinfected in 1% NaOCl for 5 min, posteriorly rinsed in sterile distilled water, and then dried with sterilized paper towels. Fruits were wounded at one point by pricking the surface with one sterile pin to a depth of 3 mm. Fruits were inoculated using the colonized agar plug (5 mm) method. Mycelial plugs were removed from the edges of 7-day-old cultures grown on PDA medium. Fruits inoculated with non-colonized agar represented the negative control. Inoculated fruits were kept in a moist chamber at 28 °C in the dark. After 48 h, the moist chamber was removed, and fruits were kept at the same temperature. Pathogenicity and aggressiveness were evaluated 8 days after inoculation. Pathogenicity was confirmed by the presence of typical anthracnose symptoms. Aggressiveness was assessed by measuring the orthogonal diameter of the lesions. The experiment was carried out with five replications per treatment (isolate), with two fruits per replicate (ten fruits/isolate), and repeated once. Differences in aggressiveness caused by *Colletotrichum* species were determined via one-way analyses of variance (ANOVAs) and means were compared by Fisher’s least significant difference (LSD) test at the 5% significance level using Statistix 9.

## 3. Results

### 3.1. Phylogenetic Reconstruction and Species Recognition

**Figure 2:**
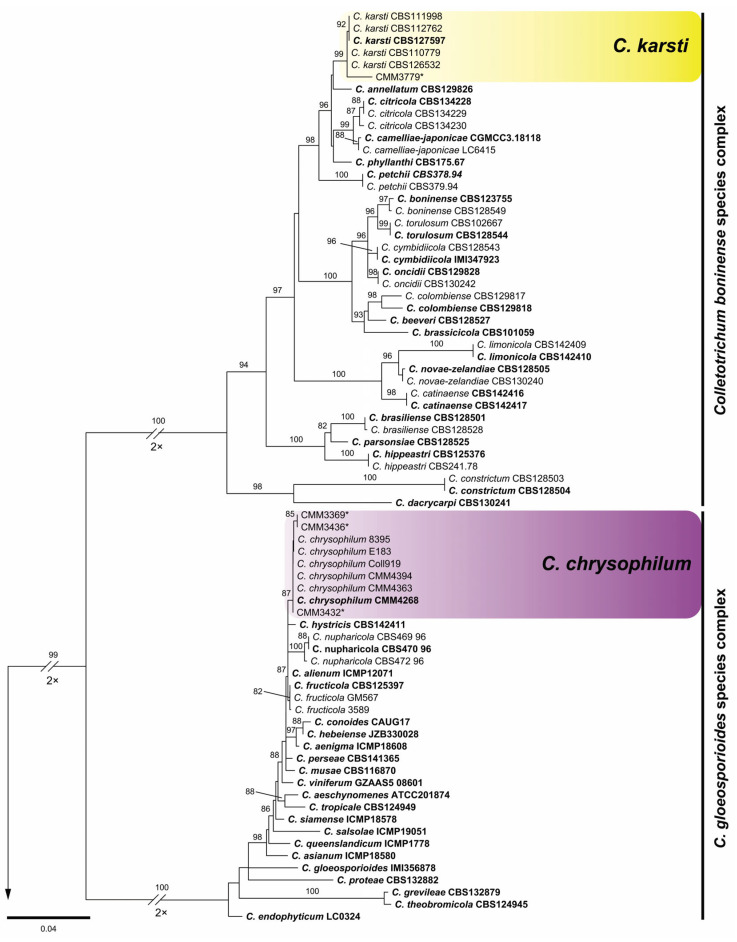

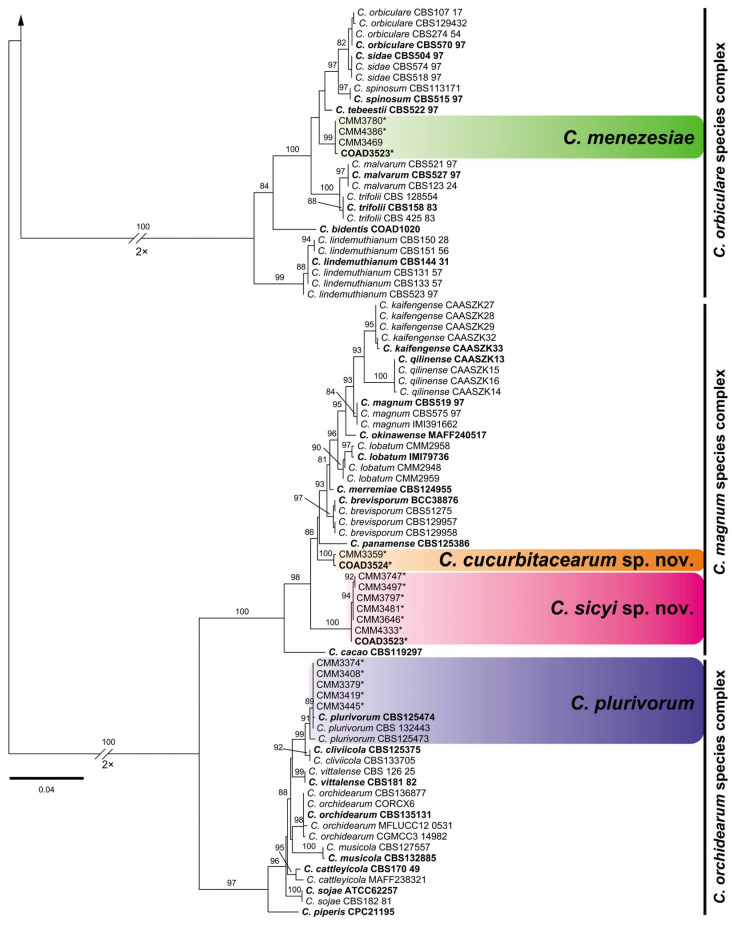
*Colletotrichum* maximum likelihood tree inferred from a concatenated alignment of *ACT*, *GAPDH*, nrITS, and *TUB*2. Isolates from the present study are marked with an asterisk “*”. Ex-types are highlighted in bold font. Significant SH-alrt bootstrap supports (≥ 80%) are shown above the branches. The scale bar indicates the estimated number of substitutions per site. The tree is rooted at the midpoint.

*Colletotrichum* isolates from chayote were distributed among 10 haplotypes according to *TUB*2 sequences. BLAST searches revealed that the isolates belong to five different *Colletotrichum* species complexes: *C. boninense sensu lato* (H1), *C. gloeosporioides s. l.* (H2, H3), *C. magnum s. l.* (H7–H10), *C. orbiculare s. l.* (H4, H5), and *C. orchidearum s. l.* (H6). The representative isolates were distributed among six species according to multilocus analysis (Figure 2) and were clearly recognized as phylogenetic species according to GCPSR criteria: H1—*C. karsti*; H2 and H3—*C. chrysophilum*; H4 and H5—*C. menezesiae*; and H6—*C. plurivorum*. These species presented significant support in the ML analysis (87–99% SH-alrt bootstrap support).

The haplotypes H7–H10 positioned within *C. magnum* s. l. were distributed in two independent clades with no sister species, which did not include the ex-type of any species previously described and presented maximum support. Moreover, these two clades were recovered as independent lineages according to GCPSR criteria. Thus, they represent novel species and are formally introduced as *C. cucurbitacearum* sp. nov. and *C. sicyi* sp. nov. in Section 3.2.

### 3.2. Taxonomy

**Figure 3:**
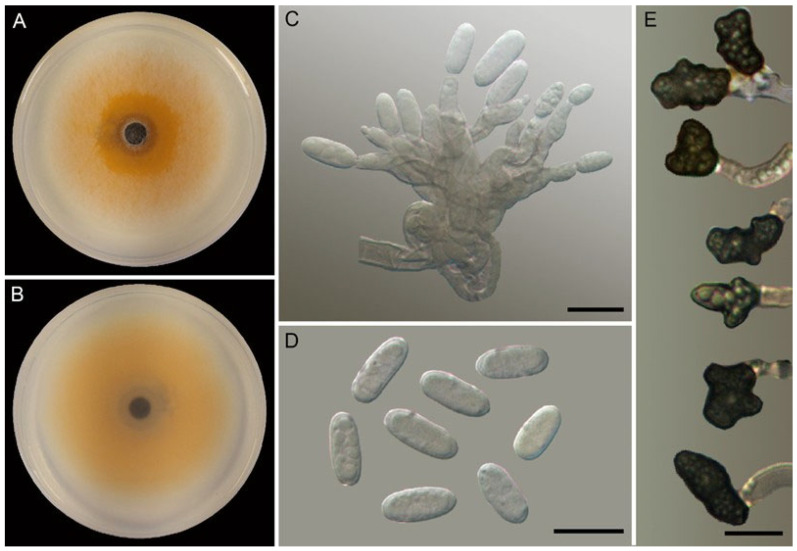
*Colletotrichum cucurbitacearum* COAD3524, ex-holotype culture. (**A**,**B**) Cultures on PDA, 7 d growth, from above (**A**) and below (**B**). (**C**) Conidiophores and conidiogenous cells. (**D**) Conidia. (**C**,**D**) From OA. (**E**) Appressoria. Scale bars: (**C**,**D**) 5 µm; (**E**) 10 µm.

*Colletotrichum cucurbitacearum* A. Reis, W.A.S. Vieira and C.A. Costa, sp. nov. Figure 3

MycoBank number: MB8556198

Systematic position: Ascomycota, Pezizomycotina, Sordariomycetes, Hypocreomycetidae, Glomerellales, Glomerellaceae.

Type: BRAZIL, SANTA CATARINA: Biguaçú, from foliar anthracnose lesions on *Sicyos edulis*, Feb 2022, A. Reis. Holotype VIC49405. Ex-type living culture COAD3524.

Etymology: Named after the host plant family, Cucurbitaceae.

Colonies on PDA had the following characteristics: luteous center with white edges from the aerial view, reverse salmon, aerial mycelium sparse, and growth rate at 25 °C 7.5–7.7 mm.dia-1 (average 7.6 ± 0.1 mm.dia-1). Conidiogenous cells on OA were hyaline, smooth-walled, and cylindrical to ampulliform. Conidiophores on OA were hyaline, smooth-walled, aseptate, unbranched, and 8.3–22.4 × 2.3–5 μm (av. 13.4 ± 3 × 3.6 ± 0.7 μm). Conidia on OA were one-celled, hyaline, smooth-walled, cylindrical with rounded ends, and 9.1–11.4 × 3.8–5.4 μm (av. 10.1 ± 0.5 × 4.7 ± 0.4 μm), with a length/width ratio of 1.8–2.7 (av. 2.2 ± 0.2). Appressoria in slide cultures were single, medium to dark brown, smooth-walled, irregular in shape, solitaries, and 3.9–10.4 × 3.6–6.7 μm (av. 7.1 ± 1.4 × 4.8 ± 0.8 μm). Setae, sclerotia, chlamydospores, and sexual morph were not observed.

Geographic distribution and host range: known from *Sicyos edulis* leaves from Biguaçú (Santa Catarina state) and Chã Grande (Pernambuco state, CMM3359), Brazil.

Notes: see *Colletotrichum sicyi* notes.

**Figure 4:**
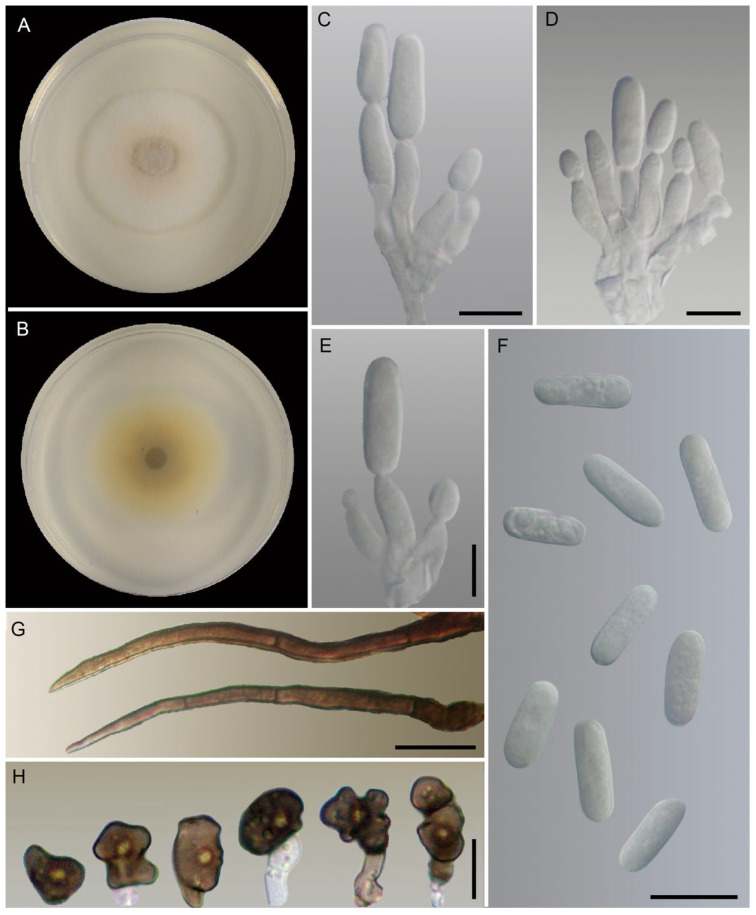
*Colletotrichum sicyi* COAD3522, ex-holotype culture. (**A**,**B**) Cultures on PDA, 7 d growth, from above (**A**) and below (**B**). (**C**–**E**) Conidiophores and conidiogenous cells. (**F**) Conida. (**G**) Setae. (**C**–**G**) From OA. (**H**) Appressoria. Scale bars: (**C**–**G**), 10 µm; (**H**), 5 µm.

*Colletotrichum sicyi* A. Reis, W.A.S. Vieira and M.P.S. Câmara, sp. nov. Figure 4

MycoBank number: MB8561199

Systematic position: Ascomycota, Pezizomycotina, Sordariomycetes, Hypocreomycetidae, Glomerellales, Glomerellaceae.

Type: BRAZIL, MINAS GERAIS: Ibirité, from foliar anthracnose lesions on *Sicyos edulis*, Feb 2022, A. Reis. Holotype VIC49403. Ex-type living culture COAD3522.

Etymology: Named after the host plant genus, Sicyos.

Colonies on PDA had the following characteristics: white with buff center from the aerial view, reverse sulfur yellow with olivaceous buff center, aerial mycelium sparse, growth rate at 25 °C 6.5–7 mm.dia-1 (average 6.7 ± 0.24 mm.dia-1). Conidiogenous cells on OA were hyaline, smooth-walled, and cylindrical to ampulliform. Conidiophores on OA were hyaline, smooth-walled, aseptate, unbranched, and 6.5–15.4× 2.1–4.2 μm (av. 10.6 ± 2.3 × 3.1 ± 0.5 μm). Conidia on OA were one-celled, hyaline, smooth-walled, cylindrical with rounded ends, and 9.1–12.1 × 3.5–4.7 μm (av. 10.4 ± 0.8 × 4.1 ± 0.3 μm), with a length/width ratio of 2.1–3.1 (av. 2.6 ± 0.3). Appressoria in slide cultures were single, medium to dark brown, smooth-walled, irregular in shape, solitaries, and 5.9–8.8 × 5–8.2 μm (av. 7.5 ± 0.7 × 6.6 ± 0.8 μm). Setae had the following characteristics: long, brown, smooth-walled, 3 septa, base truncate, tip ± acute, 36.7–54.4 × 2–3.5 μm (av. 48.7 ± 5.2 × 2.9 ± 0.4 μm). Sclerotia, chlamydospores, and sexual morph were not observed.

Geographic distribution and host range: known from *Sicyos edulis* fruit from Ibirité (Minas Gerais state, CMM3481, CMM3497), Brasília de Minas (Minas Gerais state, CMM3747, CMM4333), Brazlândia (Distrito Federal, CMM3797), and Ceilândia (Distrito Federal, CMM3646) Brazil.

Notes: *Colletotrichum cucurbitacearum* and *C. sicyi* belong to the *C. magnum* species complex and were closely positioned in the phylogenetic tree. However, these species are clearly discriminated against because they are not sisters to each other. Regarding morphology, *C*. *cucurbitacearum* presents faster growth, larger conidiophores, and smaller conidia and appressoria. In addition, setae are present only in *C. sicyi*.

### 3.3. Pathogenicity and Aggressiveness in Fruit

All six species of *Colletotrichum* were pathogenic in chayote fruit, producing lesions 8 days after inoculation. Fruit symptoms provided by all species were similar, with the development of sunken lesions, light-brown coloration, and spore mass. These symptoms were similar to those occurring in natural conditions in the field. The species were reisolated from the inoculated chayote fruits, but not from the noninoculated ones, fulfilling Koch’s postulates.

**Figure 5:**
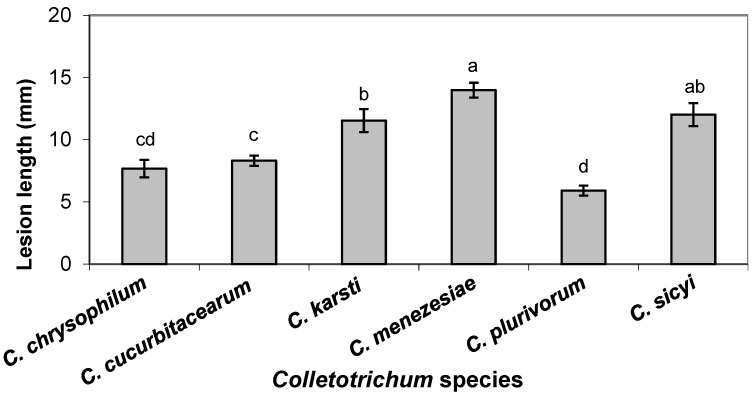
Mean lesion lengths (mm) caused by six *Colletotrichum* species associated with chayote in Brazil after inoculation of fruits with mycelium-colonized agar plugs. Bars above columns are the standard errors of the means. Columns with the same letter do not differ significantly, according to Fisher’s LSD test (*p* = 0.05).

**Figure 6:**
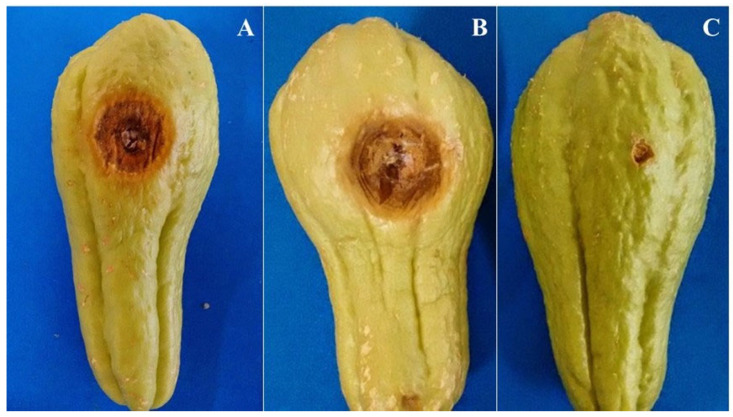
Pathogenicity of *Colletotrichum cucurbitacearum* (**A**) and *C. sicyi* (**B**) in chayote fruits. In (**C**) is the noninoculated fruit (Control).

The *Colletotrichum* species differed significantly in aggressiveness (*p* ≤ 0.05), with *C. menezesiae* and *C. sicyi* being the most aggressive species, followed by *C. karsti*. On the other hand, *C. plurivorum* was the least aggressive. *Colletotrichum chrysophilum* and *C. cucurbitacearum* showed intermediate aggressiveness and did not differ statistically according to the LSD test (*p* > 0.05) (Figure 5). Figure 6 shows the results of the pathogenicity test with one isolate each of *C. cucurbitacearum* (Figure 6A) and *C. sicyi* (Figure 6B).

## 4. Discussion

This study represents the first attempt to characterize *Colletotrichum* species associated with anthracnose of chayote in Brazil and the world using isolates from several Brazilian geographic regions and a polyphasic approach. Phylogenetic analysis showed that the 44 isolates causing anthracnose of chayote plants belonged to six different phylogenetic species. *Colletotrichum menezesiae* was recently described as a new species, infecting chayote in Brazil [39]. This study provides the first report of *C. karsti, C. chrysophilum*, and *C. plurivorum* causing disease in chayote in Brazil and worldwide and describes two new species, *C. cucurbitaciarum and C. sicyi*. This result confirms that more than one *Colletotrichum* species may infect hosts within the *Cucurbitaceae* family in addition to *C. orbiculare sensu lato*. Our results agree with other results where multiple species of *Colletotrichum* have already been found associated with different *Cucurbitaceae* species [12,13,40,41,42].

*Colletotrichum karsti* occurs in many host plants and is the most common and geographically diverse species in the *C. boninense* complex [11]. This species was first reported in China in association with anthracnose in plants belonging to Orchidaceae [43], and subsequently in cucurbits, in various ornamental plants such as Areca palm and Lotus Bamboo, and in Tobacco [11,44,45,46]. In Brazil, it has also been reported to cause anthracnose in *Passiflora edulis*, as a pathogen and endophyte in mango fruit, and, more recently, in anonaceous species [11,47,48,49,50]. *Colletotrichum chrysophilum* was recently reported as a new species from the *C. gloeosporioides* complex, causing anthracnose in banana fruits in Brazil [51]. This species was also reported later in Brazil, causing diseases in cultivated and wild cashews [52]. *Colletotrichum plurivorum* was first described as *C. sichuanensis*, causing anthracnose in *Capsicum annuum* in China’s Sichuan province, and, more recently, this species has been reported in *Phaseolus lunatus* in Brazil and papaya fruits in Japan, Taiwan, and Mexico [13,53,54,55,56].

*Colletotrichum orbiculare,* known to be an etiologic agent of anthracnose in species of the Cucurbitaceae family, was initially split into a complex with eight species: *C. lindemuthianum*, *C. malvarum*, *C. orbiculare*, *C. trifolii*, and four species pathogenic to weeds, *C. bidentis*, *C. sidae*, *C. spinosum*, and *C. tebeestii* [8]. In our paper, multilocus phylogenetic analyses revealed that some isolates belong to the *C. orbiculare* complex; however, they do not group with *C. orbiculare stricto sensu*, but to the newly described species *C. menezesiae* [39]. According to Damm et al. [8], species of the orbiculare complex are restricted to herbaceous hosts and specific to four families of plants: *Asteraceae*, *Curcubitaceae*, *Fabaceae*, and *Malvaceae*. Therefore, other studies with *C. menezesiae* are necessary to elucidate whether this rule is valid for this species.

Using a polyphasic approach, we introduce two new *Colletotrichum* species belonging to the *C. magnum* species complex in this study. Many species from the *C. magnum* complex have been found to cause anthracnose on Cucurbitaceae species worldwide. According to Damm et al. [13], the species *G. magna* (syn. *C. magnum*) was described to infect watermelon in the USA and was already reported as a pathogen of many Cucurbitaceae species such as *Cucumis melo*, squash, pumpkin (*Cucurbita maxima* and *C. moschata*), cucumber (*Cucumis sativus*), *Cucurbita pepo*, *Trichosanthes anguina*, *T. kirilowii, Lagenaria siceraria*, and *Luffa cylindrica*. Another species of this complex, *C. brevisporum*, has also been reported as a pathogen of cucurbitaceous hosts such *Sechium edule* (*Sicyos edule*), *C. pepo*, and *Momordica cochinchinensis* [57].

All the *Colletotrichum* species identified have the potential to cause anthracnose in chayote, but *C. menezesiae* and *C. sicyi* were the most aggressive species. Only one isolate of *C. karsti* was identified in our collection, but with high aggressiveness. It can be considered as a potential risk to chayote crops because it is one of the *Colletotrichum* species that is more widespread geographically and ecologically and is the most common species in the *C. boninense* complex [11,44,45,52].

The results of this study are important because they demonstrate that, surprisingly, six *Colletotrichum* species cause anthracnose in chayote and *C. orbiculare s. s.* is not the causal agent of this disease in Brazil, as has been previously reported. The precise identification of the species of *Colletotrichum* associated with anthracnose of chayote will contribute to a better understanding of the etiology and epidemiology of the disease, which will result in better decision-making in management strategies.

## Figures and Tables

**Table 1:** Primers used in this study, with sequences and references.

Genes	Primer	Sequence (5’–3’)	Reference
*ACT*	ACT-512F	ATG TGC AAG GCC GGT TTC GC	Carbone and Kohn (1999) [23]
	ACT-783R	TAC GAG TCC TTC TGG CCC AT	Carbone and Kohn (1999) [23]
*GAPDH*	GDF	GCC GTC AAC GAC CCC TTC ATT GA	Templeton et al. (1992) [24]
	GDR	GGG TGG AGT CGT ACT TGA GCA TGT	Templeton et al. (1992) [24]
ITS	ITS1	CTT GGT CAT TTA GAG GAA GTA A	White et al. (1990) [25]
	ITS4	TCC TCC GCT TAT TGA TAT GC	White et al. (1990) [25]
*TUB2*	T1	AACATGCGTGAGATTGTAAGT	O’Donnell and Cigelnik (1997) [26]
	Bt2a	GGT AAC CAA ATC GGT GCT GCT TTC	Glass and Donaldson (1995) [27]
	Bt2b	ACC CTC AGT GTA GTG ACC CTT GGC	Glass and Donaldson (1995) [27]

## Data Availability

All sequence data are available in NCBI GenBank following the accession numbers in the manuscript.

## Supplementary Materials

The following supporting information can be downloaded at https://www.mdpi.com/article/10.3390/jof10120847/s1: Table S1: Strains of *Colletotrichum* studied in this paper with details of culture collection, host, location, and GenBank accessions of the sequences.
