# Supplementary material for: Diversity of Colletotrichum Species Causing Anthracnose in Chayote in Brazil, with a Description of Two New Species in the C. magnum Complex
Source: J Fungi (Basel). 2024 Dec 9;10(12):847. doi: 10.3390/jof10120847 (PMC11676391; doi:10.3390/jof10120847)
Supplement: Supplementary file 1 [file jof-10-00847-s001.zip › jof-3233745-supplementary.pdf]

Supplementary Table S1. Strains of *Colletotrichum* studied in this paper and reference isolates used in the phylogeny, with details of culture collection, host, location and GenBank accessions of the sequences.

Colletotrichum Species Complex	*Colletotrichum* Species	Culture Number*	GenBank Accession Number			
			ACT	GAPDH	ITS	TUB2
Boninense	*C. annellatum*	CBS 129826, CH1*	JQ005570	JQ005309	JQ005222	JQ005656
Boninense	*C. beeveri*	CBS 128527, ICMP 18594*	JQ005519	JQ005258	JQ005171	JQ005605
Boninense	*C. boninense*	CBS 123755, MAFF 305972*	JQ005501	JQ005240	JQ005153	JQ005588
Boninense	*C. boninense*	CBS 128549, ICMP 15444	JQ005504	JQ005243	JQ005156	JQ005590
Boninense	*C. brasiliense*	CBS 128501, ICMP 18607, PAS12*	JQ005583	JQ005322	JQ005235	JQ005669
Boninense	*C. brasiliense*	CBS 128528, ICMP 18606, PAS10	JQ005582	JQ005321	JQ005234	JQ005668
Boninense	*C. brassicicola*	CBS 101059, LYN 16331*	JQ005520	JQ005259	JQ005172	JQ005606
Boninense	*C. catinaense*	CBS 142417 = CPC 27978	KY855971	KY856224	KY856400	KY856482
Boninense	*C. catinaense*	CBS 142416 = CPC 28019	KY855970	KY856223	KY856399	KY856481
Boninense	*C. colombiense*	CBS 129818, G2*	JQ005522	JQ005261	JQ005174	JQ005608
Boninense	*C. colombiense*	CBS 129817, G1	JQ005521	JQ005260	JQ005173	JQ005607
Boninense	*C. constrictum*	CBS 128504, ICMP 12941*	JQ005586	JQ005325	JQ005238	JQ005672
Boninense	*C. constrictum*	CBS 128503, ICMP 12936	JQ005585	JQ005324	JQ005237	JQ005671
Boninense	*C. cymbidicola*	IMI 347923*	JQ005514	JQ005253	JQ005166	JQ005600
Boninense	*C. cymbidicola*	CBS 128543, ICMP 18584	JQ005515	JQ005254	JQ005167	JQ005601
Boninense	*C. dacrycarpi*	CBS 130241, ICMP 19107*	JQ005584	JQ005323	JQ005236	JQ005670
Boninense	*C. hippeastri*	CBS 125376, CSSG1*	JQ005579	JQ005318	JQ005231	JQ005665
Boninense	*C. hippeastri*	CBS 241.78, IMI 304052	JQ005580	JQ005319	JQ005232	JQ005666
Boninense	*C. karsti*	**CMM3779**	**PP680331**	**PP680351**	**PP660978**	**PP680368**
Boninense	*C. karsti*	CBS 110779	JQ005557	JQ005296	JQ005209	JQ005643
Boninense	*C. karsti*	CBS 127597	JQ005552	JQ005291	JQ005204	JQ005638
Boninense	*C. karsti*	CBS 111998	JQ005560	JQ005299	JQ005212	JQ005646
Boninense	*C. karsti*	CBS 112762	JQ005561	JQ005300	JQ005213	JQ005647
Boninense	*C. karsti*	CBS 126532	JQ005557	JQ005296	JQ005209	JQ005643
Boninense	*C. limonicola*	CBS 142410 = CPC 31141	KY856045	KY856296	KY856472	KY856554
Boninense	*C. limonicola*	CBS 142409 = CPC 27861	KY856044	KY856295	KY856471	KY856553
Boninense	*C. novae-zelandiae*	CBS 128505, ICMP 12944*	JQ005576	JQ005315	JQ005228	JQ005662
Boninense	*C. novae-zelandiae*	CBS 130240, ICMP 12064	JQ005577	JQ005316	JQ005229	JQ005663
Boninense	*C. oncidii*	CBS 129828*	JQ005517	JQ005256	JQ005169	JQ005603
Boninense	*C. oncidii*	CBS 130242	JQ005518	JQ005257	JQ005170	JQ005604
Boninense	*C. parsonsiae*	CBS 128525, ICMP 18590*	JQ005581	JQ005320	JQ005233	JQ005667
Boninense	*C. petchii*	CBS 378.94*	JQ005571	JQ005310	JQ005223	JQ005657
Boninense	*C. petchii*	CBS 379.94	JQ005572	JQ005311	JQ005224	JQ005658
Boninense	*C. phyllanthi*	CBS 175.67, MACS 271*	JQ005569	JQ005308	JQ005221	JQ005655
Boninense	*C. torulosum*	CBS 128544, ICMP 18586*	JQ005512	JQ005251	JQ005164	JQ005598
Boninense	*C. torulosum*	CBS 102667	JQ005513	JQ005252	JQ005165	JQ005599
Gloeosporioides	*C. aenigma*	ICMP 18608*		JX010044		JX010389
Gloeosporioides	*C. aeschynomenes*	ATCC 201874*		JX009930		JX010392
Gloeosporioides	*C. alienum*	ICMP 12071*		JX010028		JX010411
Gloeosporioides	*C. chrysophilum*	CMM4268*		KX094183		KX094285
Gloeosporioides	*C. chrysophilum*	8395		KX094176		GU994473
Gloeosporioides	*C. chrysophilum*	Coll919		KX094177		KX094288
Gloeosporioides	*C. chrysophilum*	E183		KX094178		GU994472
Gloeosporioides	*C. chrysophilum*	**CMM3369**	**PP680328**	**PP680346**	**PP660971**	**PP680366**

Gloeosporioides	*C. chrysophilum*	**CMM3432**	**PP680329**	**PP680347**	**PP660983**	**PP680367**
Gloeosporioides	*C. chrysophilum*	**CMM3436**	**PP680330**	**PP680348**	**PP660984**	
Gloeosporioides	*C. conoides*	CAUG17*		KP890162		KP890174
Gloeosporioides	*C. endophytica*	MFLUCC 130418, LC0324*		KC832854		
Gloeosporioides	*C. fructicola*	CBS 125397*, ICMP 18646		JX010032		JX010409
Gloeosporioides	*C. fructicola*	1087		KX094174		KX094279
Gloeosporioides	*C. fructicola*	3589		KX094175		KX094280
Gloeosporioides	*C. fructicola*	7574				GU994471
Gloeosporioides	*C. fructicola*	GM567		JQ894630		JQ894600
Gloeosporioides	*C. gloeosporioides*	IMI 356878*, ICMP 17821, CBS 112999		JX010056		JX010445
Gloeosporioides	*C. hebeiense*	JZB 330028*		KF377495		KF288975
Gloeosporioides	*C. horii*	ICMP 10492*		GQ329681		JX010450
Gloeosporioides	*C. makassarense*	CBS 143664*		MH728820		MH846563
Gloeosporioides	*C. musae*	CBS 116870*, ICMP 19119		JX010050		HQ596280
Gloeosporioides	*C. nupharicola*	CBS 470.96*, ICMP 18187		JX009936		JX010397
Gloeosporioides	*C. perseae*	CBS141365*		KX620242		KX620341
Gloeosporioides	*C. proteae*	CBS132882*		KC297009		KC297101
Gloeosporioides	*C. queenslandicum*	ICMP 1778*		JX009934		JX010414
Gloeosporioides	*C. salsolae*	ICMP 19051*		JX009916		JX010403
Gloeosporioides	*C. siamense*	ICMP 18578*, CBS 130417		JX009924		JX010404
Gloeosporioides	*C. tainanense*	CBS 143666*		MH728823		MH846558
Gloeosporioides	*C. theobromicola*	CBS 124945*, ICMP 18649		JX010006		JX010447
Gloeosporioides	*C. tropicale*	CBS 124949*, ICMP 18653		JX010007		GU994454
Gloeosporioides	*C. viniferum*	GZAAS 5.08601*		JN412798		JN412813
Magnum	*C. cucurbitacearum* sp. nov	**CMM3359**	**PP680312**	**PP680332**	**PP660970**	**PP680352**
Magnum	*C. cucurbitacearum* sp. nov	**COAD3524***	**PP680313**	**PP680333**	**PP660974**	**PP680353**
Magnum	*C. sicyi* sp. nov.	**CMM3481**	**PP680317**	**PP680338**	**PP660986**	**PP680356**
Magnum	*C. sicyi* sp. nov.	**CMM3491**	**PP680318**	**PP680340**	**PP660987**	**PP680360**
Magnum	*C. sicyi* sp. nov.	**CMM3646**	**PP680319**	**PP680339**	**PP660988**	**PP680358**
Magnum	*C. sicyi* sp. nov.	**CMM3747**	**PP680314**	**PP680334**	**PP660977**	**PP680354**
Magnum	*C. sicyi* sp. nov.	**CMM3797**	**PP680315**	**PP680336**	**PP660980**	**PP680355**
Magnum	*C. sicyi* sp. nov.	**CMM4333**	**PP680316**	**PP680337**	**PP660981**	**PP680359**
Magnum	*C. sicyi* sp. nov.	**COAD3522***	**PP680320**	**PP680335**		**PP680357**
Magnun	*C. brevisporum*	CBS 129957	MG600966	MG600822	MG600762	MG601029
Magnun	*C. brevisporum*	CBS 129958	MG600967	MG600823	MG600763	MG601030
Magnun	*C. brevisporum*	COUFAL0009			KY021366	KY021264
Magnun	*C. brevisporum*	BCC38876*	JN050216	JN050227	JN050238	JN050244
Magnun	*C. brevisporum*	CBS512.75	MG600965	MG600821	MG600761	MG601028
Magnun	*C. brevisporum*	CMM2922	MG018380	MG018411	MG018425	MG018405
Magnun	*C. brevisporum*	CMM4732	MG436775	MG018420	MG436779	MG436777
Magnun	*C. brevisporum*	CMM2951	MG018379	MG018410	MG018434	MG018393
Magnun	*C. brevisporum*	CMM2924	MG018381	MG018412	MG018426	MG018404
Magnun	*C. cacao*	CBS 119297*	MG600976	MG600832	MG600772	MG601039
Magnun	*C. kaifengense*	CAASZK27	OL449308	OL456710	MZ475239	OL456669
Magnun	*C. kaifengense*	CAASZK28	OL449309	OL456711	MZ475240	OL456670
Magnun	*C. kaifengense*	CAASZK29	OL449310	OL456712	MZ475241	OL456671
Magnun	*C. kaifengense*	CAASZK32	OL449312	OL456714	MZ475244	OL456673

Magnun	*C. kaifengense*	CAASZK33*	OL449313	OL456715	MZ475245	OL456674
Magnun	*C. lobatum*	IMI 79736*	MG600972	MG600828	MG600768	MG601035
Magnun	*C. lobatum*	CMM2948	MG018392	MG018419	MG018428	MG018400
Magnun	*C. lobatum*	CMM2958	MG018391	MG018418	MG018433	MG018406
Magnun	*C. lobatum*	CMM2959	MG018387	MG018417	MG018422	MG018400
Magnun	*C. magnum*	CBS 519.97*	MG600973	MG600829	MG600769	MG601036
Magnun	*C. magnum*	IMI 391662	MG600975	MG600831	MG600771	MG601038
Magnun	*C. magnum*	CBS 575.97	MG600974	MG600830	MG600770	MG601037
Magnun	*C. merremiae*	CBS 124955*	MG600969	MG600825	MG600765	MG601032
Magnun	*C. panamense*	CBS 125386*	MG600970	MG600826	MG600766	MG601033
Magnun	*C. qilinense*	CAASZK13*	OL449292	OL456694	MZ475217	OL456653
Magnun	*C. qilinense*	CAASZK14	OL449293	OL456695	MZ475218	OL456654
Magnun	*C. qilinense*	CAASZK15	OL449294	OL456696	MZ475219	OL456655
Magnun	*C. qilinense*	CAASZK16	OL449295	OL456697	MZ475220	OL456656
Orbiculare	*C. bidentis*	COAD 1020*, CPC 21930	KF178578	KF178506	KF178481	KF178602
Orbiculare	*C. lindemuthianum*	CBS 144.31*	JQ005842	JX546712	JQ005779	JQ005863
Orbiculare	*C. lindemuthianum*	CBS 523.97, LARS 798	JX546623	JX546719	JX546815	JX546861
Orbiculare	*C. lindemuthianum*	CBS 150.28	JX546619	JX546715	JX546811	JX546857
Orbiculare	*C. lindemuthianum*	CBS 133.57	JX546615	JX546710	JX546807	JX546853
Orbiculare	*C. lindemuthianum*	CBS 151.56	JX546620	JX546716	JX546812	JX546858
Orbiculare	*C. lindemuthianum*	CBS 131.57	JX546615	JX546710	JX546807	JX546853
Orbiculare	*C. malvarum*	CBS 527.97*	KF178577	KF178504	KF178480	KF178601
Orbiculare	*C. malvarum*	CBS 521.97*, LARS 720, Lav-4	KF178577	KF178504	KF178480	KF178601
Orbiculare	*C. malvarum*	CBS 123.24	KF178576	KF178503	KF178479	KF178600
Orbiculare	*C. menezesiae*	**CMM3780**	**PP680326**	**PP680349**	**PP660979**	**PP680369**
Orbiculare	*C. menezesiae*	**CMM4386**	**PP680327**	**PP680350**	**PP660982**	**PP680370**
Orbiculare	*C. menezesiae*	COAD3523*	OQ704012	OQ704014	OQ701081	OQ704015
Orbiculare	*C. menezesiae*	CMM3469	OQ704011	OQ704013	OQ701080	OQ704016
Orbiculare	*C. orbiculare*	CBS 570.97*, LARS 73	KF178563	KF178490	KF178466	KF178587
Orbiculare	*C. orbiculare*	CBS 129432, USYD-2008-01	KF178566	KF178494	KF178469	KF178590
Orbiculare	*C. orbiculare*	CBS 274.54	KF178559	KF178486	KF178462	KF178583
Orbiculare	*C. orbiculare*	CBS 107.17	KF178562	KF178489	KF178465	KF178586
Orbiculare	*C. sidae*	CBS 504.97*	KF178569	KF178497	KF178472	KF178593
Orbiculare	*C. sidae*	CBS 518.97, LARS 629, Cm-4	KF178568	KF178496	KF178471	KF178592
Orbiculare	*C. sidae*	CBS 574.97, LARS 625, ATCC 96725, 3-1-1, Cm-9	KF178567	KF178495	KF178470	KF178591
Orbiculare	*C. spinosum*	CBS 515.97*, LARS 465, DAR 48942	KF178571	KF178498	KF178474	KF178595
Orbiculare	*C. spinosum*	CBS 113171, IMI 368075, STE-U 5296	KF178572	KF178499	KF178475	KF178596
Orbiculare	*C. tebeestii*	CBS 522.97*, LARS 733, 83-43	KF178570	KF178505	KF178473	KF178594
Orbiculare	*C. trifolii*	CBS 158.83*	KF178575	KF178502	KF178478	KF178599
Orbiculare	*C. trifolii*	CBS 425.83	KF178574	KF178501	KF178477	KF178598
Orbiculare	*C. trifolii*	CBS 128554, ICMP 12934, LARS 164, N85 ANW	KF178573	KF178500	KF178476	KF178597
Orchidearum	*C. cattleyicola*	CBS 170.49*	MG600963	MG600819	MG600758	MG601025
Orchidearum	*C. clivicola*	CBS 125375*	MG600939	MG600795	MG600733	MG601000
Orchidearum	*C. clivicola*	CBS 133705	MG600938	MG600794	MG600732	MG600999
Orchidearum	*C. musicola*	CBS 132885*	MG600942	MG600798	MG600736	MG601003
Orchidearum	*C. musicola*	CBS 127557	MG600943	MG600799	MG600737	MG601004
Orchidearum	*C. orchidearum*	CBS 135131*	MG600944	MG600800	MG600738	MG601005
Orchidearum	*C. orchidearum*	CBS 136877	MG600945	MG600801	MG600739	MG601006
Orchidearum	*C. piperis*	CPC 21195*	MG600964	MG600820	MG600760	MG601027

Orchidearum	*C. plurivorum*	CBS 125474*	MG600925MG600781MG600718MG600985
Orchidearum	*C. plurivorum*	CBS 125473	MG600924MG600780MG600717MG600984
Orchidearum	*C. plurivorum*	CBS 132443	MG600926MG600782MG600719MG600986
Orchidearum	*C. plurivorum*	**CMM3374**	**PP680321 PP680341 PP660972 PP680361**
Orchidearum	*C. plurivorum*	**CMM3379**	**PP680322 PP680342 PP660973 PP680362**
Orchidearum	*C. plurivorum*	**CMM3408**	**PP680323 PP680345 PP660975 PP680363**
Orchidearum	*C. plurivorum*	**CMM3419**	**PP680324 PP680343 PP660976 PP680364**
Orchidearum	*C. plurivorum*	**CMM3445**	**PP680325 PP680344 PP660985 PP680365**
Orchidearum	*C. sojae*	CBS 182.81	MG600959MG600815MG600754MG601021
Orchidearum	*C. sojae*	ATCC 62257*	MG600954MG600810MG600749MG601016
Orchidearum	*C. vittalense*	CBS 126.25	MG600941MG600797MG600735MG601002
Orchidearum	*C. vittalense*	CBS 181.82*	MG600940MG600796MG600734MG601001

* Ex-type strains. Culture numbers and GenBank accession numbers from the present study are highlited in bold font. CBS: Culture Collection of the Centraalbureau voor Schimmelcultures, Fungal Biodiversity centre, Utrecht, The Netherlands; CGMCC: China General Microbiological Culture Collection, Beijing, China; COAD: Coleção Octávio Almeida Drummond, Viçosa, Brazil; CMM: Culture Collection of Phythopathogenic Fungi "Prof. Maria Menezes", Recife, Brazil; DAR: Queensland Plant Pathology Herbarium (Australia), Queensland, Australia; ICMP: International Collection of Microorganisms from Plants, Landcare Research, Auckland, New Zealand; IMI: International Mycological Institute, CABI-Bioscience, Egham, Bakeham Lane, U.K.; MAFF: Ministry of Agriculture, Forestry and Fisheries, Tsukuba, Japan; MFLU: Mae Fah Luang University Herbarium, Thailand; NBRC: Biological Resource Center, National Institute of Technology and Evaluation, Chiba, Japan. ACT: actin; GAPDH: glyceraldehydes-3-phosphate dehydrogenase; ITS: internal transcribed spacer regions 1 & 2 including 5.8S nrDNA gene; TUB2: partial ß-tubulin. New deposited strains are indicated in bold.
